# Idiopathic Subglottic Tracheal Stenosis Misdiagnosed As Vocal Cord Dysfunction and Successfully Treated with Laser and Controlled Radial Expansion Balloon Dilation

**DOI:** 10.7759/cureus.7702

**Published:** 2020-04-16

**Authors:** Sajin M Karakattu, Karthik Vijayan, Ibrahim Haddad, Adel El Abbassi

**Affiliations:** 1 Pulmonary and Critical Care, East Tennessee State University, Johnson City, USA; 2 Internal Medicine, Quillen College of Medicine, East Tennessee State University, Johnson City, USA

**Keywords:** tracheal stenosis, pulmonary function test, vocal cord dysfunction

## Abstract

Idiopathic tracheal stenosis (ITS) is a rare condition, and diagnosis of exclusion should be suspected in patients with exercise intolerance, wheezing, and dyspnea on exertion with a flow-volume loop suggestive of fixed airway obstruction. We report a case of a 32-year-old asthmatic woman with an existing diagnosis of vocal cord dysfunction and previous normal CT scan of the neck. She continued to have fixed upper airway obstruction on repeated flow-volume loops with persistent wheezing and cough along with occasional stridor and hoarseness of voice despite appropriate management of her asthma. She was finally diagnosed with ITS on a repeat CT scan of the neck for which she underwent laser surgery, steroid injection, and controlled radial expansion balloon dilation with a successful reduction of stenosis. This case illustrates the importance of clinical suspicion for early diagnosis of ITS in poorly controlled asthmatic patients and the relevance of non-surgical management of this condition.

## Introduction

Idiopathic tracheal stenosis (ITS) is usually misdiagnosed as asthma, which delays appropriate management and adds to the frustrations of patients. Diagnosis is based on history, neck imaging, and spirometry and confirmed with direct visualization of the stenotic segment during bronchoscopy. Treatment approaches include surgical and non-surgical options.

## Case presentation

A 32-year-old Caucasian woman, a lifelong non-smoker with a body mass index of 44.3, was referred to our outpatient pulmonary clinic for asthma and vocal cord dysfunction. She had complaints of cough, wheezing, and dyspnea on exertion with occasional stridor for many years for which she was on a steroid inhaler along with albuterol inhaler. She reported poor control of her symptoms and denied any history of recurrent infections, increased sputum production, hemoptysis, esophageal reflux, tracheal trauma, previous endotracheal intubation, or any inflammatory or infiltrative processes. Her erythrocyte sedimentation rate, antinuclear antibody, rheumatoid factor, anti-myeloperoxidase (MPO), anti-proteinase 3 (PR3), anti-Sjögren's syndrome A (SSA/Ro), anti-Sjögren Syndrome B (SSB/La), anti-Scl70, and circulating levels of alpha -1 antitrypsin were within the normal limits. She had no eosinophilia, and her IgE level was normal, along with normal immunoglobulin levels. She also had a sleep study, which was normal, and over 18 months, had four pulmonary function tests (PFTs), all of which showed an obstructive pattern with no bronchodilator response and normal diffusion capacity. Flow-volume loops showed flattening of inspiratory and expiratory maneuvers, suggestive of fixed upper airway obstruction.

**Figure 1:**
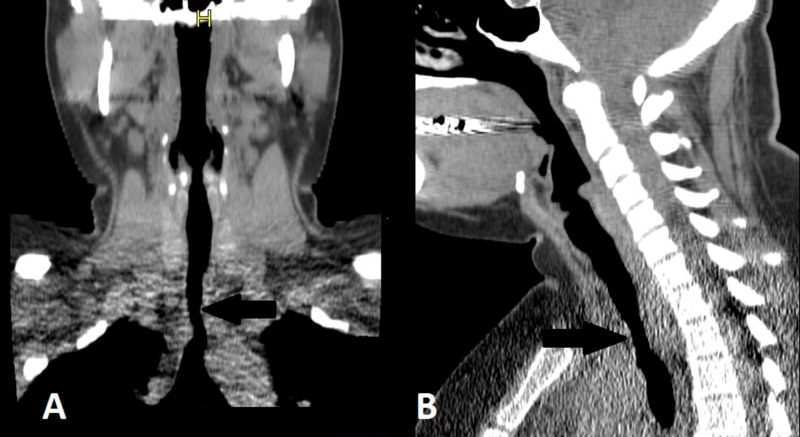
Coronal (A) and sagittal (1B) sections showing 60% tracheal narrowing approximately 6 cm below the vocal cords (arrows).

Three years prior to our initial evaluation, she had a CT scan of the neck, which showed only mild prominence of parapharyngeal mucosa that appeared reactive with no other abnormalities and underwent otorhinolaryngology evaluation and was diagnosed as vocal cord dysfunction. During one of her follow-up appointments in our clinic, she endorsed new onset hoarseness of voice. Giving her new onset hoarseness of voice and multiple previous PFTs suggestive of fixed upper airway obstruction, we decided to repeat her imaging. She underwent a CT scan of the neck, which confirmed an irregularly narrowed (60%) trachea 3 cm above the carina and 6 cm below the vocal cords with septation along the right posterior aspect of the trachea (Figure 1).

**Figure 2:**
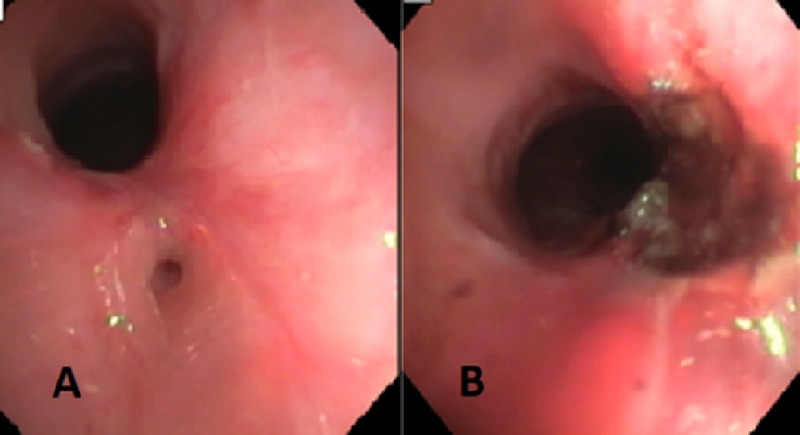
Complex narrowing that caused a significant scarring and a small airway (A). Post laser laster therapy and controlled radial expansion balloon dilation with a relief of stenosis (B).

Subsequently, she underwent bronchoscopy that showed complex tracheal stenosis approximately 3 cm above the carina and extending for 4 cm into the trachea. It showed complex narrowing that caused significant scarring in the middle leading to a small airway on the right posterior wall of the trachea, giving an appearance of a tracheal bronchus (Figure 2A). The narrowest area was 4 cm above the carina. She had laser surgery followed by controlled radial expansion (CRE) balloon dilation of tracheal stenosis, which reached an internal diameter of 14 mm with complete resolution of stenosis confirmed with follow-up bronchoscopy (Figure 2B). She followed up in our clinic a few weeks later and endorsed complete resolution of wheezing, dyspnea, hoarseness of voice, and cough. However, her flow-volume loop continued to show fixed airway obstruction even after six months of her procedure.

## Discussion

ITS represents a diagnosis of exclusion and occurs exclusively in middle-aged women, mostly postmenopausal [1-3]. It produces stenosis of 1-3 cm, which is densely collagenous, and unaccompanied by systemic disease [4-6]. An association between gastroesophageal reflux disease and laryngotracheal stenosis has been found. However, a definite conclusion regarding its role has not yet been reached, especially since the stenosis does not typically progress [6,7]. The female predominance suggests hormonal etiology of the disease. One of the postulated mechanisms is the absence of estrogen receptors at the site of stenosis, which leads to an increase in fibroblast growth factor resulting in the formation of the stenotic lesion; however, investigations failed to show direct correlation, and the cause of the disease has remained elusive. Persistent mucosal inflammation and altered microbial composition termed "microbiota dysbiosis" are the hallmark of the disease; however, the relationship between tracheal microbiota dysbiosis and ITS is not well understood [8,9].

Tracheal stenosis could be due to extrinsic (direct external compression as in mediastinal malignancy) or intrinsic causes. Etiologies of intrinsic stenosis include autoimmune conditions (systemic lupus erythematosus, sarcoidosis, amyloidosis, granulomatosis with polyangiitis, polychondritis, and Wegener's granulomatosis), inhalational burn, infectious processes (rhinolaryngoscleroma, tuberculosis, bacterial tracheitis, diphtheria, and histoplasmosis), or idiopathic as in our patient [8]. The diagnosis is based on the combination of findings on history, physical exam, and diagnostic testing. Presenting symptoms are shortness of breath, cough, stridor, hoarseness, and wheezing, and patients are often misdiagnosed with asthma with minimal responsiveness to steroid and bronchodilator inhalers. Spirometry, CT scan, and bronchoscopy are necessary modalities to diagnose this disorder [10]. The flow-volume loop on spirometry reveals flattening of both the inspiratory and expiratory loops, despite numerical data showing no definite obstruction. CT scan of the neck will show the extent and severity of stenosis, but confirmation of the diagnosis requires direct visualization with bronchoscopy.

There is some controversy regarding the most favored approach for the treatment of ITS, with balloon dilation being the most conservative approach [11]. Other modalities such as airway stenting, laser treatment (Nd-YAG laser surgery via a fibreoptic bronchoscope), endoluminal spray cryotherapy, tailored cricoplasty, and surgical reconstruction or resection are all valid options [12-15]. Approximately 47%-71% of the patients with a non-malignant disease may be managed with balloon dilation alone, although the recurrence rate after balloon dilation is higher than other approaches [16]. In cases where ITS involves only the upper trachea, segmental resection with end-to-end anastomosis can be performed. It is usually done as one-stage resection and reconstruction, most often including a portion of the lower larynx with more than 90% of patients preserving their voice quality [17]. In a case series of 263 patients diagnosed with idiopathic subglottic stenosis and treated with laryngotracheal resection and reconstruction, the overall success rate was reported to be 91%, considering all forms of recurrences [18]. Medical treatment with agents such as mitomycin C and steroid injections are used as adjunctive treatments.

## Conclusions

ITS is a rare condition, and a diagnosis of exclusion should be suspected in patients who present with unexplained wheezing, hoarseness, dyspnea on exertion, and cough along with flow-volume loops suggestive of fixed upper airway obstruction. It may mimic symptoms of asthma and, in our case, an existing diagnosis of vocal cord dysfunction, which in turn can lead to delay in diagnosis and definitive management. Despite growing evidence of lesser recurrence rates in favor of surgical correction, we believe that there is still a significant role of minimally invasive measures in the treatment of the stenosis. However, it is agreeable that repeated laser and CRE balloon dilations would render the patient an unsuitable candidate for definitive surgical management. Patients may continue to have flow-volume loops suggestive of fixed upper airway obstruction despite clinical improvement, which makes it more challenging to follow up these patients for relapse. More studies regarding the resolution of the fixed defect in the flow-volume loop after laser surgery and balloon dilation are needed.
